# The Characteristics of PD-L1 Inhibitors, from Peptides to Small Molecules

**DOI:** 10.3390/molecules24101940

**Published:** 2019-05-20

**Authors:** Yanwen Zhong, Xuanyi Li, Hequan Yao, Kejiang Lin

**Affiliations:** Department of Medicinal Chemistry, China Pharmaceutical University, Nanjing 210009, China; yhysjysypt@163.com (Y.Z.); 15261483658@163.com (X.L.)

**Keywords:** programmed cell death ligand protein 1, pharmacophore, peptide, small molecule

## Abstract

The programmed cell death ligand protein 1 (PD-L1) is a member of the B7 protein family and consists of 290 amino acid residues. The blockade of the PD-1/PD-L1 immune checkpoint pathway is effective in tumor treatment. Results: Two pharmacophore models were generated based on peptides and small molecules. Hypo 1A consists of one hydrogen bond donor, one hydrogen bond acceptor, two hydrophobic points and one aromatic ring point. Hypo 1B consists of one hydrogen bond donor, three hydrophobic points and one positive ionizable point. Conclusions: The pharmacophore model consisting of a hydrogen bond donor, hydrophobic points and a positive ionizable point may be helpful for designing small-molecule inhibitors targeting PD-L1.

## 1. Introduction

Under normal circumstances, the immune system can identify and remove tumor cells in the tumor microenvironment [1]. However, to survive and grow, tumor cells can adopt different strategies to escape from the immune system. Immune checkpoints such as CTLA-4 (cytotoxic T lymphocyte-associated antigen-4) and PD-1 (programmed cell death protein 1), which regulate the activation of lymphocytes and balance immune responses, can protect tumor cells from the immune response. Immune checkpoint inhibitors, as one of focus of tumor immunotherapy, can be targeted in the immune system instead of tumor cells to stimulate an immune response [2,3]. Programmed cell death 1 (PD-1) is one of the best-studied immune checkpoints [4].

PD-1 is a member of the B7 superfamily which consists of 288 amino acid residues and acts as an inhibitory receptor. PD-1 is one of the death receptors which have been identified as a subgroup of the tumor necrosis factor (TNF)-receptor superfamily, which can induce apoptosis via a conserved cytoplasmic signaling module called the death domain, including TNF-R1, Fas, DR3 (death receptor 3) and so on [5,6]. PD-L1 and PD-L2 are the two ligands of PD-1 which are expressed on immune cells such as NK (natural killer) cells, active T cells and B cells [7]. The programmed cell death ligand protein 1 (PD-L1) is a member of the B7 protein family and consists of 290 amino acid residues. The PD-1/PD-L pathway plays a crucial role in immunotherapy. The binding of PD-1 and PD-L1 or PD-L2 results in the phosphorylation of the immune receptor tyrosine-based inhibition motif and the immune receptor tyrosine-based switch motif, which can recruit phosphatases SHP (Src homology 2 domain-containing tyrosine phosphatase)-1 and SHP-2 to the PD-1 intracellular domain; the phosphatases from the SHP family are mainly responsible for the effect caused by PD-1 intracellularly. After the phosphorylation of the SHP family, the downstream signaling pathways of T-cell receptors such as the phosphoinositide 3-kinase (PI3K)/Akt pathway will be inhibited, leading to the inhibition of the activity and proliferation of T cells. The binding of PD-1 and ligands will also result in a decrease in phosphorylation of the CD3ζ (cluster of differentiation 3ζ) chains and ZAP-70 (Zeta-associated protein-70) [8]. This process can be blocked through the use of PD-1 or PD-L1 inhibitors [9,10].

Inhibitors of PD-1 may lead to the blockade of both the PD-1/PD-L1 pathway and the PD-1/PD-L2 pathway. However, inhibitors of PD-L1 can only block the PD-1/PD-L1 pathway, not the PD-1/PD-L1 pathway. Compared to PD-1 inhibitors, PD-L1 inhibitors can reduce the incidence of side effects resulting from immune disorders [11,12,13]. The FDA has approved three humanized monoclonal IgG4 antibodies targeting PD-L1, Atezolizumab, Avelumab and Durvalumab [14]. In addition to their great success in clinical trials, the problems of mAbs are very obvious, including higher production costs, lower oral bioavailability, poor tumor penetration, immune-related adverse events, etc. [15,16]. Moreover, compared to peptides and small molecules, the immunogenicity of mAbs can result in severe immune-related adverse events (irAEs) in a few cases. Due to the long half-lives and strong target occupancy of mAbs, the target inhibition is sustained, and irAEs are intractable [14]. In comparison with monoclonal antibodies, small-molecule and peptide inhibitors targeting PD-L1 have smaller molecular weights and more controllable pharmacokinetic and pharmacological profiles [17]. However, the development of small-molecule inhibitors of the PD-1/PD-L1 pathway is slow; only a few small-molecule and peptide inhibitors have been reported. In 2016, CA-170 became the only small-molecule inhibitor targeting PD-L1 in phase I clinical trials [18,19]. AUNP-12 (Aurigene NP-12) is the first peptide targeting PD-L1. Compared to peptides, small molecules have advantages in terms of their oral and plasma stability. Moreover, the oral bioavailability of small molecules is higher, and the synthesis of small molecules is easier [17,20]. The study of small-molecule PD-L1 inhibitors has attracted attention; because of the complexity and plasticity of the PD-L1 surface, it is difficult to design active small-molecule inhibitors targeting PD-L1. Therefore, many efforts have been made to develop small-molecule inhibitors, but only a few small-molecule inhibitors have been reported and patented [21,22].

In 2015, the crystal holo-structure of hPD-1 (human PD-1) with hPD-L1 (human PD-L1) was solved by the team of Zak (Protein Data Bank (PDB) ID: 4ZQK). This result resolved the uncertainty brought by the mPD-1 (mouse PD-1)/hPD-L1 crystal structure [23]. The crystal structure shows the interaction between PD-1 and PD-L1, in which three hydrophobic regions are thought to be major hot spots on the interaction surface of PD-L1. The discovery of the crystal structure of PD-1/PD-L1 provides a basis for designing non-antibody-based inhibitors of PD-L1. In 2016, three classes of peptide inhibitors of PD-L1 were published by Bristol–Myers–Squibb (BMS). Crystal structures of these peptides and PD-L1 were reported by Zak in 2017 (PDB ID: 5O45 and 5O4Y) [24], which can be helpful in designing peptide inhibitors targeting PD-L1. In 2016, a series of small-molecule inhibitors of PD-L1 was discovered by scientists at BMS. The team of Krzysztof M. Zak studied the interaction between these compounds and PD-L1 and provided a series of crystal structures of PD-L1 and its small-molecule ligands (PDB ID: 5J89, 5J8O, 5N2D, 5N2F, 5NIU, 5NIX) [25,26,27]. These crystal structures show that the interaction of PD-1/PD-L1 was blocked by the small molecules, which induced PD-L1 dimerization, and then the interaction surface of PD-1 could be occupied by another PD-L1 protein [25]. This information may be beneficial in designing small-molecule inhibitors targeting PD-L1.

It is challenging to design small-molecule inhibitors targeting the surface in protein–protein interactions because of the flexibility of proteins [28,29]. The disclosure of the crystal structures of PD-1/PD-L1 provides the possibility of designing small-molecule inhibitors targeting PD-L1 via computer-aided drug design (CADD). In this study, we built two pharmacophore models based on the crystal structures of peptides and small molecules, respectively (Supplementary Materials). Two models were compared to investigate the characteristics of PD-L1 inhibitors. These characteristics of peptides and small molecules are important in future efforts to discover and optimize PD-L1 inhibitors.

## 2. Results

### 2.1. Pharmacophore Model Hypo 1A Hypotheses and Validation

We built seven pharmacophore models based on the crystal structure of peptide-71 and PD-L1 (Table 1).

**Figure 1:**
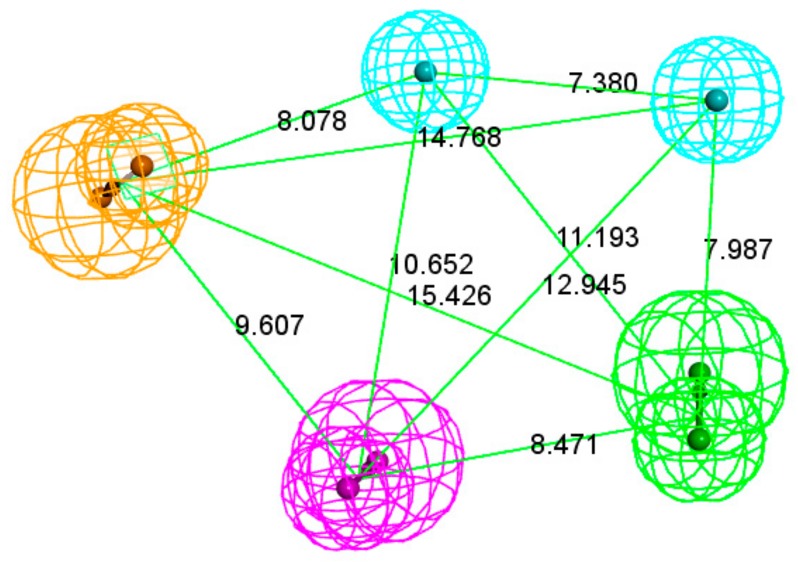
Pharmacophore model Hypo 1A. As the figure shows, the aromatic ring point is orange, the hydrophobic point is blue, the hydrogen bond acceptor is green, and the hydrogen bond donor is purple. The distance between the pharmacophore features is reported in angstroms.

Based on the selectivity score, sensitivity and specificity, Pharmacophore03 is the best choice. Pharmacophore03 is chosen as the best mainly because it seems that it is more important to effectively remove the inactive compound; in the case of the current example, most PD-L1 inhibitors are macromolecules. Compared to Pharmacophore06, Pharmacophore03 not only predicts active small molecules but also has good predictive power for active peptides, although the two pharmacophores have the same specificity and sensitivity to small-molecule active compounds. Pharmacophore03 was selected as the pharmacophore model (Hypo 1A) for the peptide targeting of PD-L1. Hypo 1A consists of one hydrogen bond donor, one hydrogen bond acceptor, two hydrophobic points and one aromatic ring point (Figure 1).

### 2.2. Decoy Set of Hypo 1A

The specificity and sensitivity were calculated to evaluate the quality of the generated model. The resulting sensitivity was 0.741, and the specificity was 0.993 (Table 2).

### 2.3. ROC Curve of Hypo 1A

**Figure 2:**
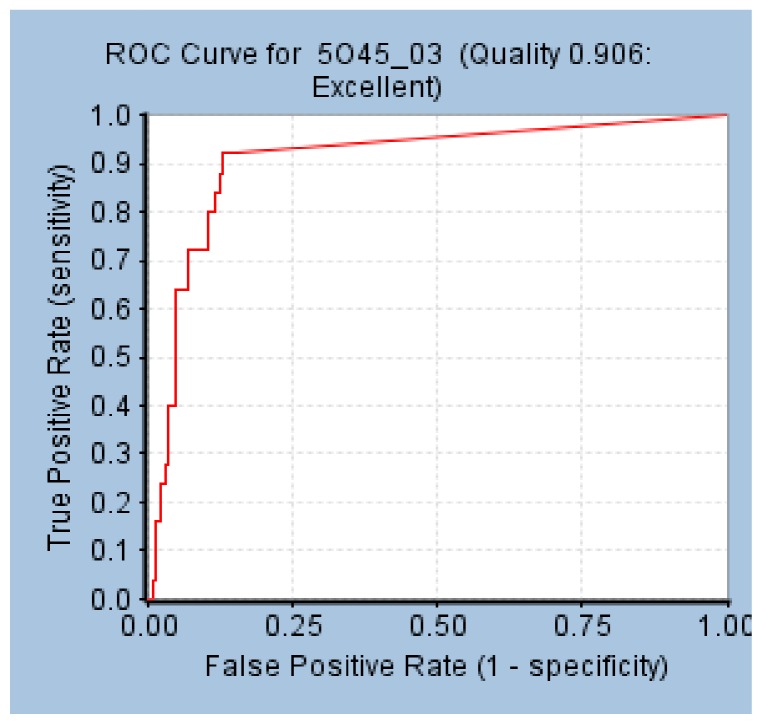
Receiver operating characteristics (ROC) curve of Hypo 1A.

The AUC value of the model was 0.906, and Hypo 1A was thought to have the ability to distinguish active molecules from inactive molecules (Figure 2).

### 2.4. Pharmacophore Model Hypo 1B Hypotheses and Validation

We built 10 pharmacophore models based on the crystal structure of BMS-1001 and PD-L1. The fit value is a predictor of the activity of the compound and reflects the degree of matching of the compound to the pharmacophore model (Table 3).

**Figure 3:**
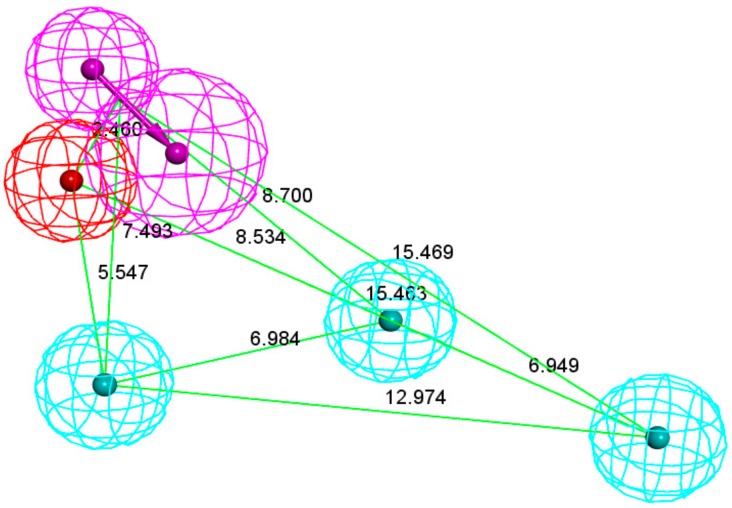
Pharmacophore model 1B. As the figure shows, the positive ionizable point is red, the hydrophobic point is blue, and the hydrogen bond donor is purple.

Model02 was selected as the pharmacophore model (Hypo 1B) with the highest correlation. Hypo 1B consists of one hydrogen bond donor, three hydrophobic points and one positive ionizable point (Figure 3).

### 2.5. Decoy Set of Hypo 1B

Specificity and sensitivity were calculated to evaluate the quality of the generated model Hypo 1B. The resulting sensitivity was 0.709, and the specificity was 1 (Table 4).

### 2.6. ROC Curve of Hypo 1B

**Figure 4:**
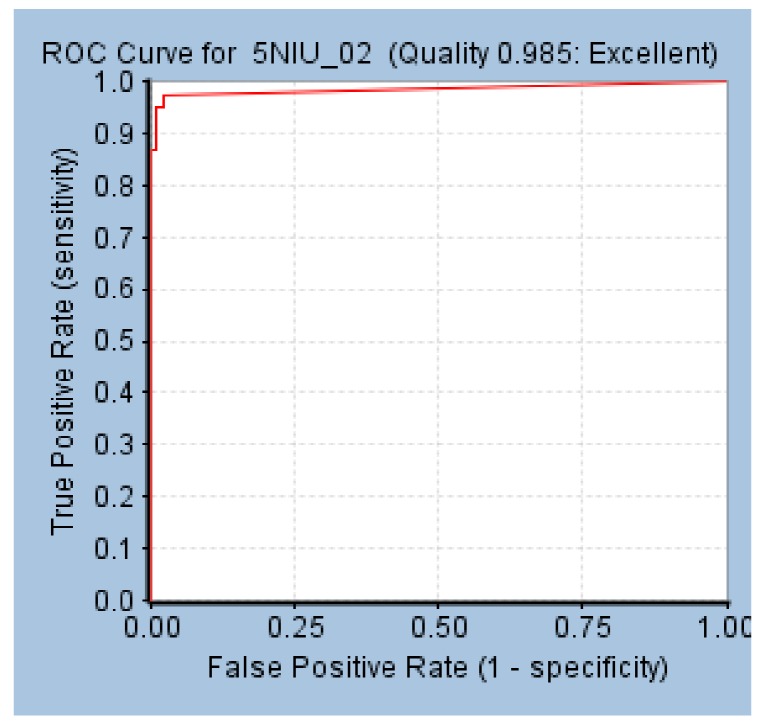
ROC curve of Hypo 1B.

The AUC value of the model was 0.985, and Hypo 1B was thought to have the ability to distinguish active molecules from inactive molecules (Figure 4).

### 2.7. Molecular Docking Study

We docked BMS compounds to PD-L1 (PDB ID: 5NIX) based on CHARMM using CDOCKER in DS. The interaction energy of BMS-1166, BMS-1001, BMS-37, BMS-202, BMS-8, BMS-200, BMS-242 and PD-L1 were 76.614, 67.602, 65.688, 63.355, 59.252, 70.329, 65.587 respectively.

## 3. Materials and Methods

The computational molecular modelling studies were carried out using Discovery Studio (DS, Accelrys, San Diego, CA, USA).

### 3.1. Generation and Validation of the Pharmacophore Model Based on Peptides

**Figure 5:**
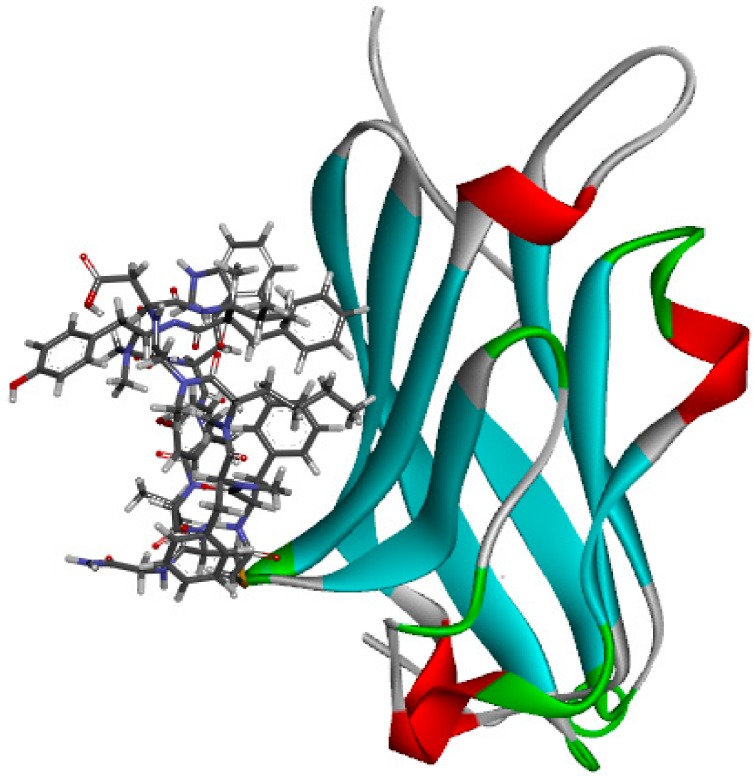
The crystal structure of peptide-71 and human programmed cell death ligand 1 (hPD-L1) (Protein Data Bank (PDB) ID: 5O45).

Since the crystal structures of PD-1/PD-L1 were published, more and more peptides targeting PD-L1 have been disclosed. Generating pharmacophore models based on peptide inhibitors can be helpful in designing non-antibody inhibitors of PD-L1. The structures of two macrocyclic peptides targeting PD-L1 disclosed by BMS were published in 2017 [24], of which peptide-57 contains 15 residues (PDB ID:5O4Y) and peptide-71 contains 14 residues (PDB ID:5O45). Pharmacophore models based on peptides were generated using the crystal structure of peptide-71 and PD-L1 (PDB ID: 5O45) because the IC_50_ of peptide-71 is 7 nM, indicating that it is more active than peptide-57 (Figure 5). The “receptor–ligand pharmacophore generation” protocol was used to identify a set of features from the binding ligand. Features of the ligands were identified by receptor–ligand interactions, and hydrogen bond acceptors, hydrogen bond donors, hydrophobic points, negative ionizable points, positive ionizable points and aromatic rings were considered during pharmacophore generation. A maximum of five features was permitted in each pharmacophore. The “maximum pharmacophores” protocol was set to 10, while the “maximum features” and “minimum features” protocols were set to 5. Seven pharmacophore models were generated.

The decoy set, ROC (receiver operating characteristics) curve, sensitivity and specificity were used to evaluate the quality of the pharmacophore hypothesis. Sensitivity is defined as the power of a model to identify positives, and specificity is defined as the power of a model to determine negatives. These attributes were calculated as follows to validate the pharmacophore model:

Sensitivity = true positives/(true positives + false negatives)

Specificity = true negatives/(true negatives + false positives)

The decoy consists of positives and negatives. The 148 angiotensin-converting enzyme inhibitors used as negatives were selected from the database (http://dude.docking.org/) at random. The 27 active compounds used as positives were acquired from Integrity [30] or Reaxys [31]. The “build 3D database” protocol was applied to build the database, and the “search 3D database” protocol was applied to screen the database. The “build 3D database” protocol was used to generate the ligand database, which was indexed via sub-structures, pharmacophore features and shape information for database searching via the “search 3D database” protocol.

Active and inactive molecules were used to generate the ROC curve, which is used to evaluate the ability of a pharmacophore model to distinguish active molecules from inactive molecules. The area under the curve (AUC) value is the area under the ROC curve, which often ranges from 0 to 1. The model is thought to be better if the AUC value is closer to 1.

### 3.2. Generation and Validation of the Pharmacophore Model Based on Small Molecules

**Figure 6:**
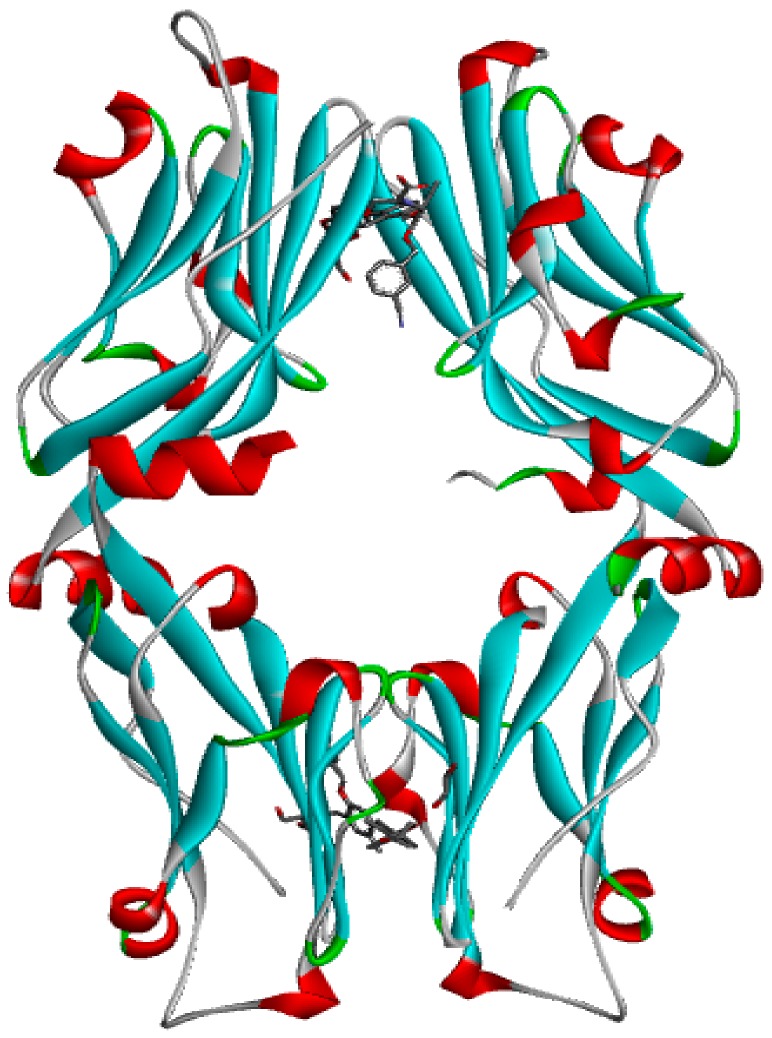
The crystal structure of Bristol–Meyers–Squibb (BMS)-1001 and hPD-L1 (PDB ID: 5NIU).

Scientists at Bristol–Myers–Squibb (BMS) have discovered a series of nonpeptidic small-molecule PD-L1 inhibitors, and the activities of these compounds were tested in a homogeneous time-resolved fluorescence (HTRF) binding assay [25,26,27]. In this study, the crystal structure of BMS-1001 and hPD-L1 (PDB ID: 5NIU) was chosen to generate and evaluate the inhibitor pharmacophore (Figure 6). The “receptor–ligand pharmacophore generation” protocol was used to identify a set of features from the binding ligand. A maximum of five features was permitted in each pharmacophore. Hydrogen bond acceptors, hydrogen bond donors, hydrophobic points, negative ionizable points, positive ionizable points and aromatic rings were selected for pharmacophore generation. The “maximum pharmacophores” protocol was set to 10, while the “maximum features” and “minimum features” protocols were set to 5. Ten pharmacophore models were generated.

The decoy set and ROC curve were used to evaluate the quality of the pharmacophore hypothesis. The decoy set including 260 molecules was constructed from 150 negatives, which were angiotensin-converting enzyme inhibitors selected from the DUDE (A Database of Useful Decoys Enhanced) database at random, and 110 active molecules selected from Reaxys.

In addition to BMS-1001, which was used to generate the pharmacophore models, the BMS series contains six other small molecules: BMS-8, BMS-37, BMS-200, BMS-202, BMS-242, and BMS-1166. Additionally, 6 other small molecules with known activity values were selected in Reaxys (Table 5). “Ligand Pharmacophore Mapping” protocol was employed to screen 13 small molecules, in which “maximum omitted features” protocol was set to 0 and the “fitting method” protocol was set to Rigid. The “Fit Value” was used to measure how well the ligands fit the pharmacophore model. The ligands fit the model better when the fit value was higher.

### 3.3. Molecular Docking Study

According to reports [23], the hydrophobic pocket consisting of Tyr56, Glu58, Arg113, Met115, Tyr123 of PD-L1 is the optimal binding site for small molecules. The crystal structure of PD-L1 was downloaded from the Protein Data Bank (PDB). Water molecules were deleted and hydrogen atoms were added in the protein. The “clean protein” protocol was applied to prepare the protein. The “prepare ligands” protocol was applied to prepare BMS compounds. The space located at (−8.651, 60.227, −19.21) with a radius of 12 Angstrom was defined as the binding site. CDOCKER (a representative docking method in Discovery Studio) with default settings was used to dock the compounds to the protein based on the CHARMM (Chemistry at HARvard Macromolecular Mechanics) forcefield.

### 3.4. Analysing Interactions between PD-L1 and BMS Compounds

The crystal structures of PD-L1 and BMS-1166 (PDB ID: 5NIX), PD-L1 and BMS-1001 (PDB ID: 5NIU), PD-L1 and BMS-200 (PDB ID: 5N2F), PD-L1 and BMS-37 (PDB ID: 5N2D), PD-L1 and BMS-202 (PDB ID: 5J89), and PD-L1 and BMS-8 (PDB ID: 5J8O) were downloaded from the Protein Data Bank (PDB). Water molecules were deleted, and hydrogen atoms were added to the protein. The “display receptor–ligand interactions” and “analyze ligand poses” protocols were used to analyze the interactions between PD-L1 and the BMS compounds.

### 3.5. Comparing the Model Based on Peptides with the Model Based on Small Molecules

The “pharmacophore comparison” protocol was used to map and align the two models generated based on peptides and small molecules, respectively. The root mean squared error (RMSE) is an indicator of matching pharmacophore features.

## 4. Discussion

### 4.1. Features of Hypo 1A

**Figure 7:**
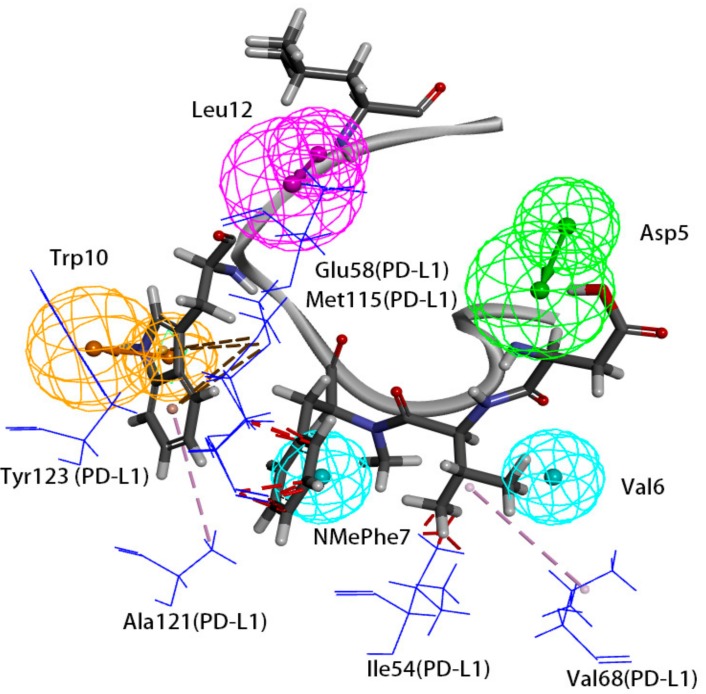
Superposition between Hypo 1A and peptide-71. As the figure shows, the hydrophobic point is blue, the hydrogen bond donor is purple, the hydrogen bond acceptor is green, and the aromatic ring point is orange. The interacted residues of PD-L1 are blue.

**Figure 8:**
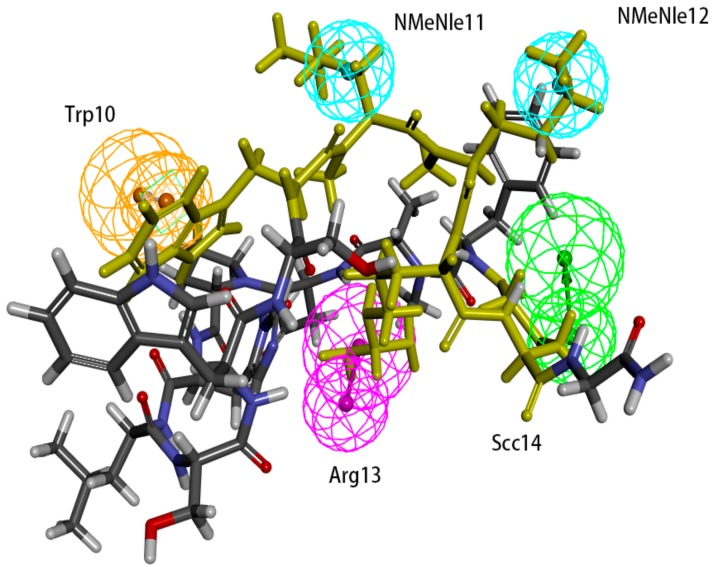
Superposition between Hypo 1A and peptide-57. As the figure shows, the hydrophobic point is blue, the hydrogen bond donor is purple, the hydrogen bond acceptor is green, and the aromatic ring point is orange. The labelled residues are yellow.

Hypo 1A consists of one hydrogen bond donor, one hydrogen bond acceptor, two hydrophobic points and one aromatic ring point. The hydrophobic points were located at NMePhe7 and Val6, the aromatic ring point was located at Trp10, the hydrogen bond donor was located at Leu12, and the hydrogen bond acceptor was located at Asp5 (Figure 7). Peptide-57 can be well mapped with Hypo 1A. The two hydrophobic points were located at NMeNle12 and NMeNle11, the aromatic ring point was located at Trp10, the hydrogen bond donor was located at Arg13, and the hydrogen bond acceptor was located at Scc14 (Figure 8). The hydrophobic zone on peptide-57 consists of Phe1, Trp8, Trp10, NMeNle11 and NMeNle12. According to the report, if each of the residues responsible for interactions is replaced by a smaller amino acid, the activity will drop. The activity will drop from 9 nm to 3656 nm if Trp10 is lacking. Analyzing the interactions between PD-L1 and peptide-57, we can observe that residues located at the Arg13 and Scc14 provided solvent contact points [24]. The activity of peptide may not drop drastically if Arg13 and Scc14 are replaced by other hydrophilic amino acids.

We can observe that Leu12 on peptide-71 interacts with Glu58 on PD-L1 via hydrogen bonds, and an intramolecular hydrogen bond can be generated between Asp5 and Tyr8 on peptide-71. Val6 on peptide-71 interacts with Ile54 on PD-L1 via hydrophobic bonds, Trp10 on peptide-71 interacts with Ala121 on PD-L1 via hydrophobic bonds, and NMePhe7 on peptide-71 interacts with Met115 on PD-L1 via hydrophobic bonds. Intramolecular hydrogen bonds can be generated between Arg13 and Trp8 and between Scc14 and Ser7 on peptide-57. Trp10 on peptide-57 interacts with Arg113, Met115, and Tyr123 on PD-L1 via hydrophobic bonds. In conclusion, Ile54, Arg113, Met115, Ala121, and Try123 of PD-L1 may be important in the hydrophobic interactions between peptides and PD-L1.

The binding surface of the peptide consists of hydrophobic regions and hydrophilic regions, and the hydrophobic interactions are essential for the binding of peptides to PD-L1 [24]. According to a report, the affinity between PD-L1 and peptide-71 is dominated by several interactions with low energy in shallow pockets instead of in any noticeable pockets [24,32]. The hydrophobic interaction is the major type of interaction involved in the binding of PD-L1 and peptide-71. The hydrophobic area consists mostly of Phe1, NMePhe7, and Trp10, supplemented with NMeNle3 and Val6. Phe1, Trp10 and Val6 were related to the hydrophobic interaction between peptide-71 and Tyr56, Ala121, and Ile54 on PD-L1, respectively. Moreover, Leu12 and Scc13 contributed to the formation of hydrogen bonds between peptide-71 and Glu58, Asp61, and Asn63 of PD-L1. The binding of PD-L1 and peptide-57 was also mainly guided by hydrophobic interactions. The hydrophobic pocket on the surface of PD-L1, which was filled with Trp10 on peptide-57, consists of Tyr56, Glu58, Arg113, Met115, and Tyr123. Moreover, NMeNle12 and NMeNle11 were related to weak hydrophobic interactions between peptide-57 and PD-L1. Additionally, Leu6 and Trp8 provided two hydrogen bonds with PD-L1. It is consistent with our pharmacophore that Ile54, Arg113, Met115, Ala121, and Try123 of PD-L1 are related to the hydrophobic interactions between peptides and PD-L1.

### 4.2. Features of Hypo 1B

**Figure 9:**
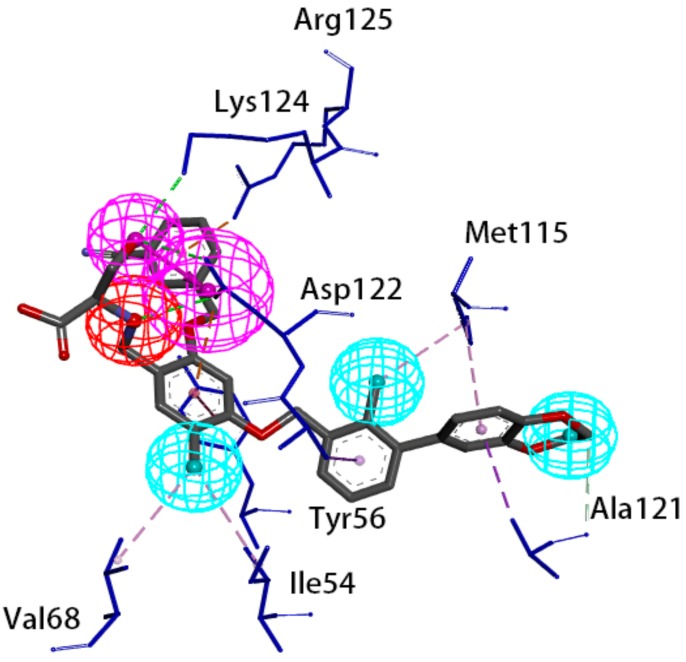
Superposition between BMS-1001 and the pharmacophore model. As the figure shows, the positive ionizable point is red, the hydrophobic point is blue, and the hydrogen bond donor is purple. The interacted residues of PD-L1 are blue.

The model consists of one hydrogen bond donor, three hydrophobic points and one positive ionizable point. According to the interactions between PD-L1 and its ligand, we can observe that the group of the ligand corresponding to the hydrophobic features interacts with Ile54, Tyr56, Val68, Met115 and Ala121 of PD-L1 via hydrophobic interactions, and the group of the ligand corresponding to the hydrogen bond donor interacts with Asp122 and Lys124 of PD-L1 via hydrophobic interactions. In addition, the group corresponding to the positive ionizable point interacts with Asp122 (Figure 9). The outcome concluded from the pharmacophore model is consistent with the conclusion from the analysis of interactions between PD-L1 and BMS compounds.

By analyzing the interactions between PD-L1 and BMS compounds, we observed that Ile54, Tyr56, Val68, Met115 and Ala121 of PD-L1 are important in generating hydrophobic bonds between PD-L1 and molecules; that Asp122 and Lys124 of PD-L1 are helpful in forming hydrogen bonds between molecules and PD-L1; and that the positive ionizable located at Asp122 of PD-L1 may be essential for the interactions between molecules and PD-L1. This outcome was consistent with Hypo 1B, which consists of one hydrogen bond donor, three hydrophobic points and one positive ionizable point.

**Figure 10:**
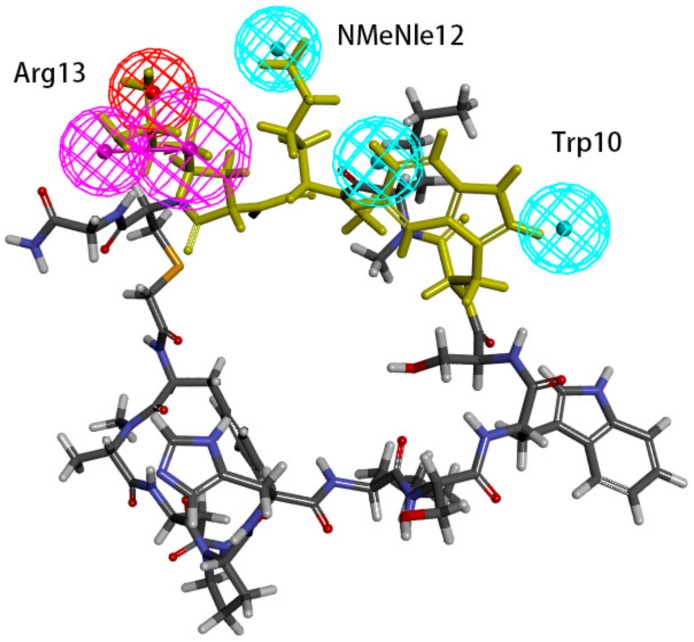
Superposition between Hypo 1B and peptide-57. As the figure shows, the positive ionizable point is red, the hydrophobic point is blue, and the hydrogen bond donor is purple. The labelled residues are yellow.

**Figure 11:**
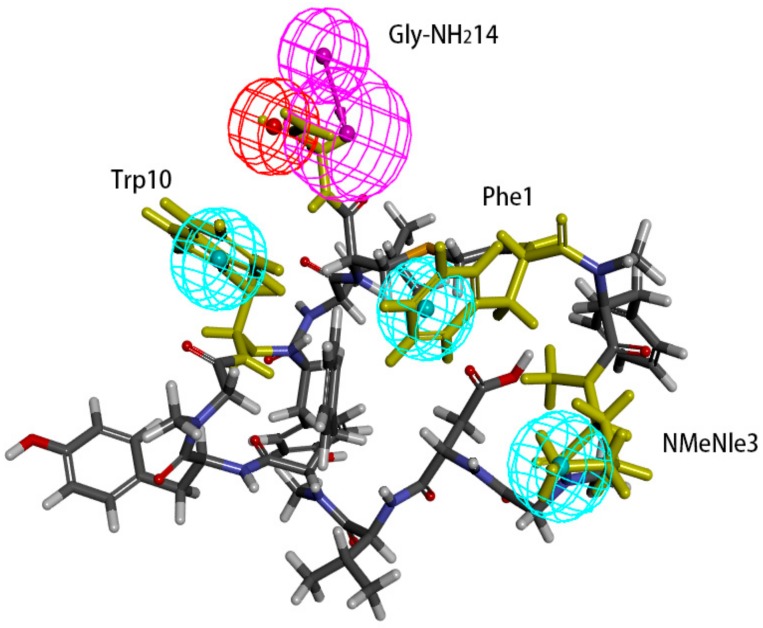
Superposition between Hypo 1B and peptide-71. As the figure shows, the positive ionizable point is red, the hydrophobic point is blue, and the hydrogen bond donor is purple. The labelled residues are yellow.

An attempt was made to map peptide-57 and peptide-71 with Hypo 1B. Peptide-57 could be well mapped with the model (Figure 10). The positive ionizable point was located at Arg13, and the hydrogen bond donor was also located at Arg13. The three hydrophobic points were located at NMeNle12 and Trp10. Peptide-71 could be mapped with the pharmacophore model, except for a positive ionizable point (Figure 11). The hydrogen bond donor was located at Gly-NH_2_14, and the three hydrophobic points were located at Phe1, NMeNle3, and Trp10.

### 4.3. Comparison between Hypo 1A and Hypo 1B

**Figure 12:**
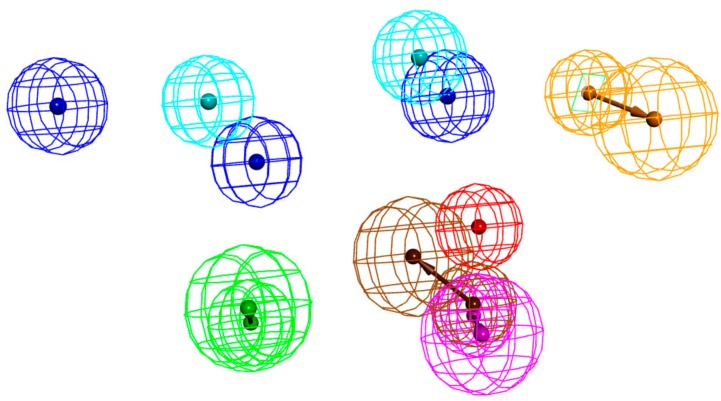
Superimposition of Hypo 1A and Hypo 1B. As the figure shows, in Hypo 1A, the hydrophobic point is light blue, the hydrogen bond acceptor is green, the hydrogen bond donor is purple, and the aromatic ring point is orange. In Hypo 1B, the hydrophobic point is Lyons blue, the positive ionizable point is red, and the hydrogen bond donor is brown.

The two models were superimposed using the pharmacophore comparison in DS, and the RMSE of Hypo 1A and 1B was 2.58. There were some common features between the two models; two hydrophobic points and one hydrogen bond donor could almost be matched. However, a hydrophobic point and a positive ionizable point of Hypo 1B and an aromatic ring point and a hydrogen bond acceptor of Hypo 1A were not in the same location (Figure 12).

**Figure 13:**
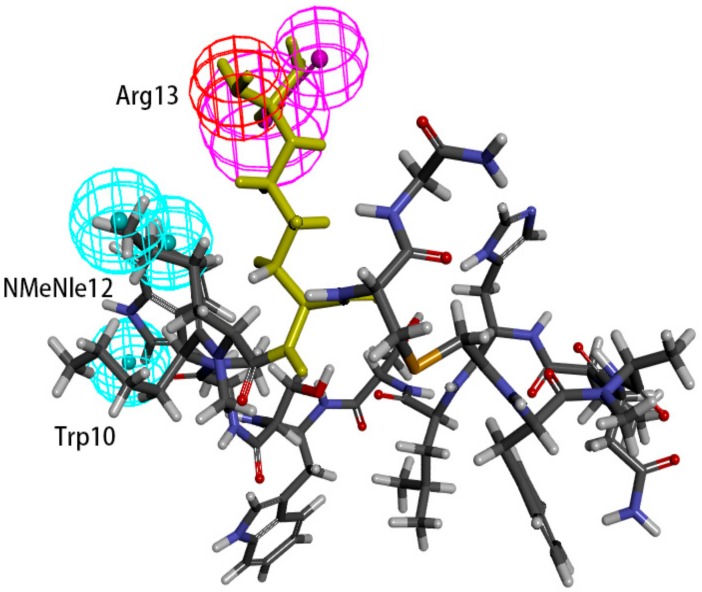
Superposition between Hypo 1B and peptide-57. As the figure shows, the positive ionizable point is red, the hydrophobic point is blue, and the hydrogen bond donor is purple. The yellow amino acid is Arg13.

There were some differences between Hypo 1A and Hypo 1B. First, a hydrophobic point of Hypo 1B and an aromatic ring point of Hypo 1A were not in the same location. The aromatic ring point of Hypo 1A located at Trp10 of peptide-71 interacted with Met115 and Ala121 of PD-L1 via hydrophobic interactions, and it was thought to play the same role as that of the hydrophobic point [24]. The hydrophobic and aromatic ring features of the two models were accommodated in the same hydrophobic pocket on PD-L1, which consists of Ile54, Tyr56, Met115, Ala121, and Tyr123. Second, the hydrogen bond acceptor did not exist in Hypo 1B. Residues located at the hydrogen bond acceptor provided solvent contact points [24] and didn’t play a key role in the interaction between PD-L1 and ligands, so the hydrogen bond acceptor may be unnecessary in Hypo 1B. Third, the positive ionizable point was non-existent in Hypo 1A. The superposition between Hypo 1B and two peptides showed that the positive ionizable point matched peptide-57 well, of which the IC_50_ value is 9 nM. When peptide-57 was mapped with Hypo 1B, the positive ionizable point was located at Arg13 (Figure 13). This outcome meant that a positive ionizable point may be necessary for both small-molecule and peptide inhibitors. Though differences between the two models did exist, the model generated based on small-molecule inhibitors was more representative and may be helpful in the design of non-antibody-based PD-L1 inhibitors.

## 5. Conclusions

In this study, the critical chemical features of PD-L1 inhibitors were found via pharmacophore models. Two pharmacophore models, Hypo 1A and Hypo 1B, were built based on small molecules and peptides, respectively. Hypo 1A consists of two hydrogen bond donors, three hydrophobic points and one positive ionizable point. Hypo 1B consists of one hydrogen bond donor, one hydrogen bond acceptor, two hydrophobic points and one aromatic ring point. The reliability of the pharmacophore models was validated by ROC curves and a decoy set. Hydrophobic features located in the same hydrophobic pocket are essential for both peptide and small-molecule inhibitors. The hydrogen bond donors of two models could be mapped. Though the positive ionizable point only exists in the pharmacophore model based on small-molecule inhibitors, it may be important for both small-molecule and peptide inhibitors. The similarity between Hypo1A and Hypo1B means that pharmacophore models consisting of a hydrogen bond donor, a hydrophobic point and a positive ionizable point may be helpful in designing small-molecule inhibitors targeting PD-L1. 

## Figures and Tables

**Table 1:** Seven generated pharmacophore models.

Pharmacophore	Number of Features	Feature Set	Selectivity Score	Sensitivity *	Specificity *
Pharmacophore01	5	DHHHR	8.8639	0.703	0.993
Pharmacophore02	5	ADHHR	8.8639	0.703	0.919
Pharmacophore03	5	ADHHR	8.8639	0.741	0.993
Pharmacophore04	5	ADHHR	8.8639	0.667	0.980
Pharmacophore05	5	ADHHR	8.8639	0.889	0.966
Pharmacophore06	5	ADHHR	8.8639	0.741	0.993
Pharmacophore07	5	AHHHR	7.9504	0.778	0.838

* Sensitivity = true positives/(true positives + false negatives), specificity = true negatives/(true negatives + false positives).

**Table 2:** Results of the decoy set.

Parameter	Values
The number of molecules in the database	175
The number of actives in the database	27
The number of hit molecules from the database	21
The number of active molecules in the hit list	20
False negatives	6
False positives	1
% Yield of actives	74.1
% Sensitivity	74.1
% Specificity	99.3

**Table 3:** Compound experimental IC_50_ values and predicted fit values of 10 pharmacophore models.

Compounds	BMS-1166	BMS-1001	BMS-202	28131141	BMS-200	28131140	BMS-242	BMS-37	28131145	28131143	R *
IC_50_ (nM)	1.4	2.25	18	43	80	6-100	6–100	6–100	110–1000	110–1000	
Model01 Fit Value	3.81036	4.48433	none	3.71533	3.80462	2.80741	none	none	1.56344	none	0.655
Model02 Fit Value	4.16447	3.93019	2.68308	3.7521	2.98747	2.73383	2.6811	2.67567	3.04527	2.58668	0.819
Model03 Fit Value	4.42391	4.52196	3.53526	3.90221	3.99688	3.30513	3.6466	3.95454	4.30285	4.01664	0.565
Model04 Fit Value	4.14129	4.63203	1.71529	2.9739	4.17607	2.10849	2.35671	1.76556	4.33115	2.77859	0.47
Model05 Fit Value	4.1626	4.4831	3.1851	3.27722	3.38762	3.0444	3.51356	2.73352	3.67542	3.56603	0.781
Model06 Fit Value	4.12881	4.49658	1.69069	3.43773	3.9225	2.71941	1.94917	1.82323	3.79388	2.59189	0.529
Model07 Fit Value	4.5101	4.72431	2.82402	3.79522	2.07746	3.38077	3.46818	3.61272	3.98516	3.93575	0.539
Model08 Fit Value	4.47768	4.73352	3.58699	4.03414	3.35641	3.77544	4.08634	4.03562	3.35984	4.07816	0.679
Model09 Fit Value	3.82049	4.05292	0.09738	4.00387	4.45517	2.82319	1.18001	0.17939	2.31305	0.11979	0.428
Model10 Fit Value	3.30267	4.09233	1.80806	3.41994	4.28178	2.67542	2.27937	1.80307	3.69411	2.88655	0.28

* The correlation coefficient (R) is a numerical measure of the correlation between experiments and predictions.

**Table 4:** Results of the decoy set.

Parameter	Values
The number of molecules in the database	260
The number of actives in the database	110
The number of hit molecules from the database	78
The number of active molecules in the hit list	78
False negatives	32
False positives	0
% Yield of actives	70.9
% Sensitivity	70.9
% Specificity	100

**Table 5:** Structures of compounds showing IC_50_ values determined via the homogenous time-resolved fluorescence (HTRF) binding assay.

Name	Structure	IC_50_ (nM)
BMS-1166	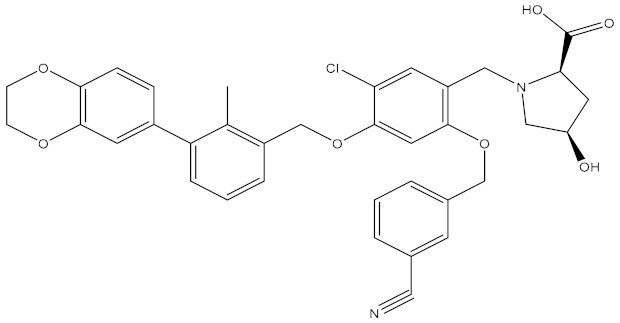	1.4
BMS-1001	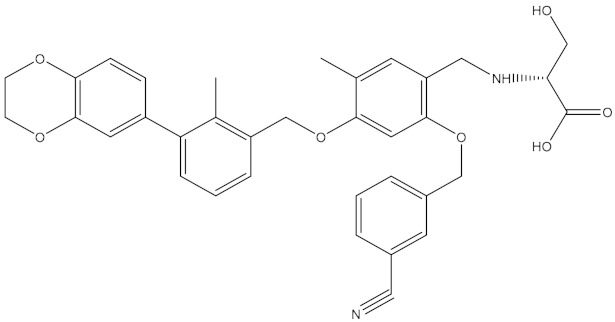	2.25
BMS-202	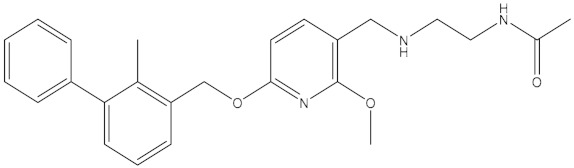	18
28131141	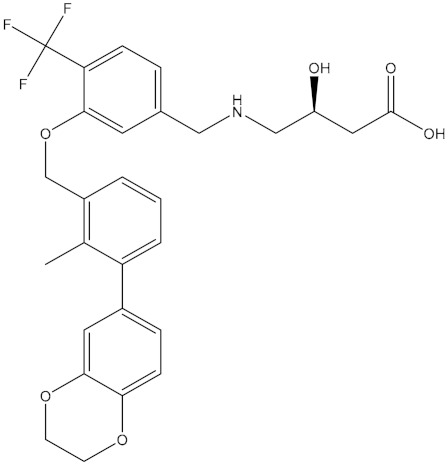	43
BMS-200	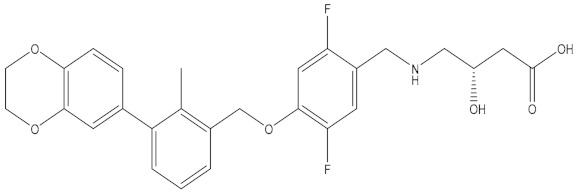	80
28131140	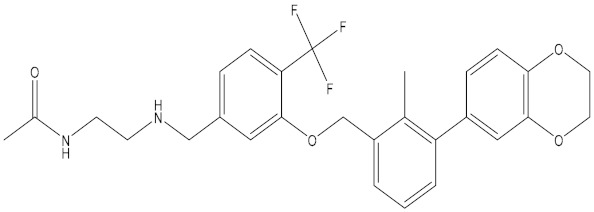	6-100
BMS-242	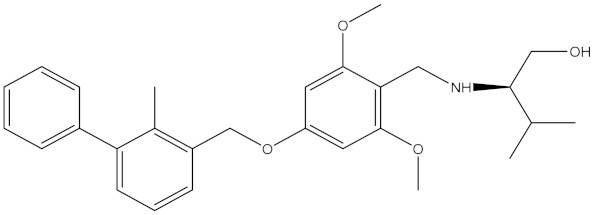	6-100
BMS-37	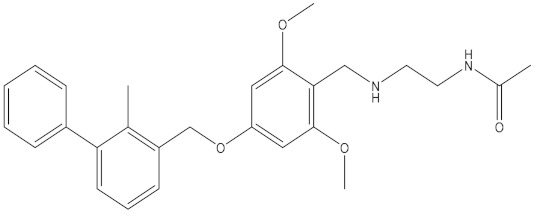	6-100
28131135	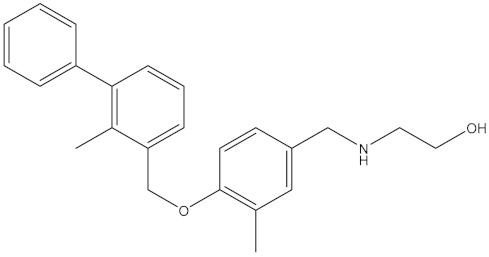	6-100
28131138	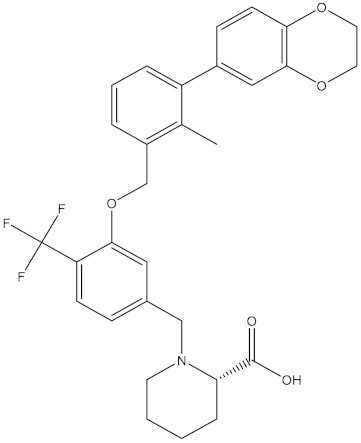	6-100
BMS-8	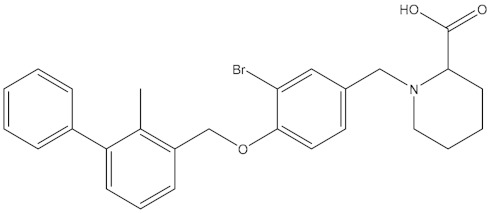	146
28131145	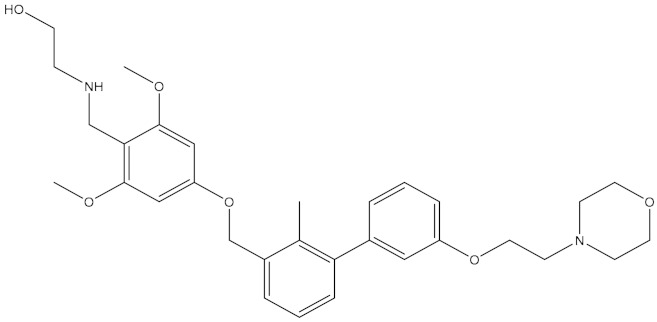	110-1000
28131143	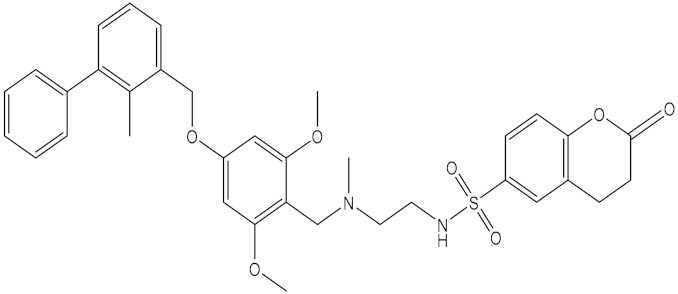	110-1000

## Supplementary Materials

The supplementary materials are available online.
